# Using Reactome to build an autophagy mechanism knowledgebase

**DOI:** 10.1080/15548627.2020.1761659

**Published:** 2020-06-02

**Authors:** Thawfeek Mohamed Varusai, Steven Jupe, Cristoffer Sevilla, Lisa Matthews, Marc Gillespie, Lincoln Stein, Guanming Wu, Peter D’Eustachio, Emmanouil Metzakopian, Henning Hermjakob

**Affiliations:** a European Molecular Biology Laboratory, European Bioinformatics Institute (EMBL-EBI), Wellcome Genome Campus, Cambridge, UK; b Open Targets, Wellcome Genome Campus, Cambridgeshire, UK; c COSMIC, Wellcome Sanger Institute, Wellcome Genome Campus, Hinxton, Cambridge CB10 1SA, UK; d Department of Biochemistry and Molecular Pharmacology, NYU Langone Medical Center, New York, NY, USA; e Ontario Institute for Cancer Research, Toronto, ON, Canada; f Pharmaceutical Sciences, St.John’s University, Queens, NY, USA; g Department of Molecular Genetics, University of Toronto, Toronto, Canada; h Department of Medical Informatics and Clinical Epidemiology, School of Medicine, Oregon Health and Science University, Oregon, Portland, OR, USA; i UK Dementia Research Institute, University of Cambridge, Cambridge Biomedical Campus, Cambridge, UK; j State Key Laboratory of Proteomics, Beijing Proteome Research Center, Beijing Institute of Radiation Medicine, National Center for Protein Sciences, Beijing, China

**Keywords:** Annotation, autophagy, biocuration, curation, enrichment analysis, knowledgebase, mechanistic analysis, molecular reactions, pathways, Reactome

## Abstract

The 21st century has revealed much about the fundamental cellular process of autophagy. Autophagy controls the catabolism and recycling of various cellular components both as a constitutive process and as a response to stress and foreign material invasion. There is considerable knowledge of the molecular mechanisms of autophagy, and this is still growing as new modalities emerge. There is a need to investigate autophagy mechanisms reliably, comprehensively and conveniently. Reactome is a freely available knowledgebase that consists of manually curated molecular events (reactions) organized into cellular pathways (https://reactome.org/). Pathways/reactions in Reactome are hierarchically structured, graphically presented and extensively annotated. Data analysis tools, such as pathway enrichment, expression data overlay and species comparison, are also available. For customized analysis, information can also be programmatically queried. Here, we discuss the curation and annotation of the molecular mechanisms of autophagy in Reactome. We also demonstrate the value that Reactome adds to research by reanalyzing a previously published work on genome-wide CRISPR screening of autophagy components.

**Abbreviations:** CMA: chaperone-mediated autophagy; GO: Gene Ontology; MA: macroautophagy; MI: microautophagy; MTOR: mechanistic target of rapamycin kinase; SQSTM1: sequestosome 1

## Introduction

The major intracellular degradation system in eukaryotes, known as autophagy, is achieved by delivering unwanted cytoplasmic cargo to the lysosome for recycling. This vital process is triggered by various stimuli, including starvation, stress and infection. The canonical autophagy mechanism involves the formation of an autophagy membrane (phagophore) around target cytoplasmic material to form the autophagosome. The next step is the fusion of the autophagosome with the lysosome to form the autolysosome. The enclosed material is degraded, returning cellular nutrients back to the cytoplasm.

This seemingly simple autophagic process is actually quite intricate, involving several components, modulators and alternate mechanisms. To add further complexity, proteins involved in the autophagic machinery and their regulation play similar roles in several cellular mechanisms, including apoptosis, secretion, and endocytic pathways, thereby linking all of these processes at the molecular level. Autophagic proteins are involved in development and are implicated in various pathologies, such as cancer, neurodegeneration and immune diseases [1]. All these factors make it difficult to comprehensively and reliably study the autophagy mechanism. There are several open questions in the field regarding inter-pathway crosstalk, non-canonical mechanisms, and regulating stimuli [2].

To facilitate effective research, there is a need for a comprehensive and reliable resource that presents all the available information on autophagy mechanisms. There have been some efforts in the community to accumulate and present knowledge in this way. The “Autophagy Database”, created by the National Institute of Genetics, provides information on autophagy protein structures [3]. The Human Autophagy Database (HADb) lists human genes/proteins involved in the autophagy machinery (http://autophagy.lu/). The Gene Ontology (GO) team have curated detailed components of the autophagy process [4]. However, to the best of our knowledge, there is no resource that informs researchers about the mechanistic significance of autophagy proteins via reactions and pathways.

It is impossible to understand or even study such a complex process as autophagy without being able to access the intricacies of the mechanisms, including interactors, reactions and pathway crosstalk. Reactome is a free, manually curated knowledge base that addresses this need by providing a user-friendly platform to access and analyze information on molecular mechanisms (https://reactome.org/) [5]. The sophisticated infrastructure of Reactome efficiently captures fine molecular details of the mechanism and allows inference of crosstalk with other pathways. We present a systematic and detailed landscape of autophagy machinery in Reactome. Curated and annotated information for interactions and reactions are provided in a user-friendly style. To accommodate different user preferences, information is presented graphically, hierarchically, and as text. There are several pathway analysis tools available that allow users to investigate available curated data or user-defined empirical data. Reactome aims to be a one-stop service for researchers, providing comprehensive and updated molecular information on autophagic reactions and pathways along with analysis tools.

## Results

### Evolving autophagy knowledge in Reactome

**Figure 1.**
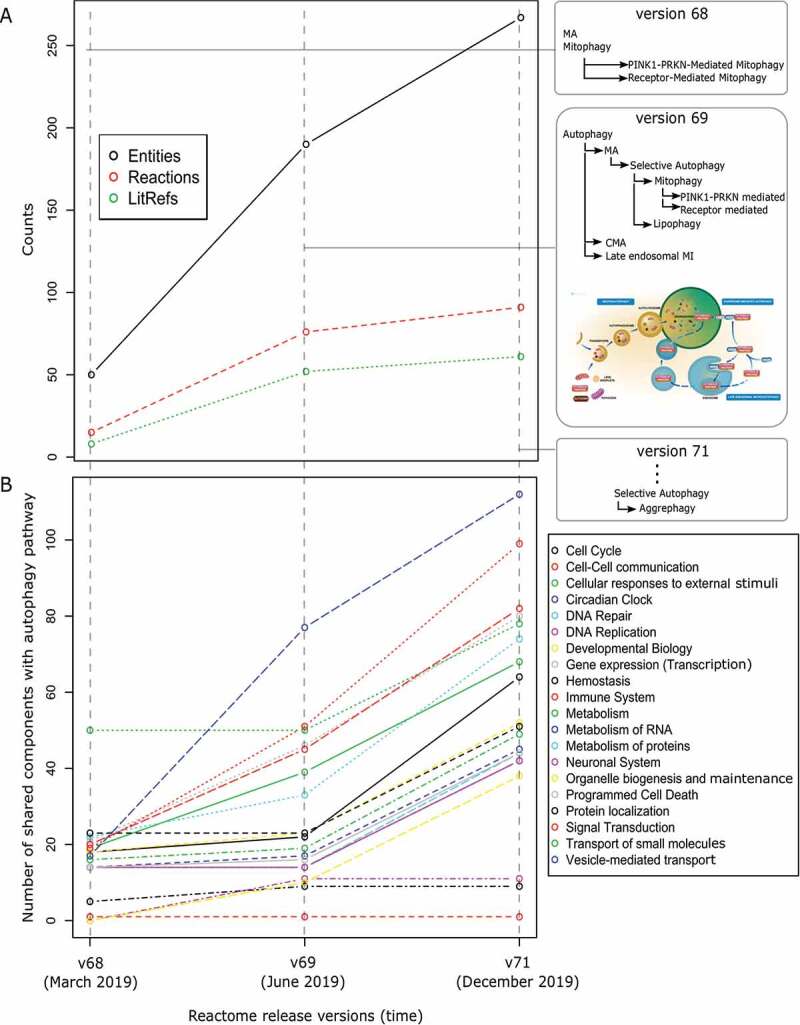
Evolving autophagy knowledge in Reactome. (A) Growing number of components, reactions and literature references curated in the autophagy pathway in different Reactome release versions (modifications of the pathway hierarchy in each release are shown in the right panel). (B) Increased interaction of autophagy components with other pathways with the inclusion of new knowledge

A systematic survey of scientific literature was carried out to curate molecular mechanisms of autophagy. Curation of autophagy pathways was initiated in late 2018 and the number of components, reactions and associated literature references have increased steadily over time (Figure 1A). Consequently, annotations of the interaction of autophagy components with other pathways have grown, with some pathways sharing more interactions than others (Figure 1B). Furthermore, we have created a new top-level pathway for autophagy with a robust hierarchical structure that allows easy information updates. Together, data in Reactome shows a positive trend of knowledge assimilation in autophagy mechanisms with newer releases.

### Reactome as an effective platform to host autophagy mechanisms

The Reactome data model is quite complex and allows the recording of various types of biological information. Consequently, Reactome serves as a robust platform to host mechanistic details of cellular events allowing for expansion and revision. For comparison, we considered another resource that has comprehensively curated the autophagy process – GO [4]. An overview of the curated information and annotated relations in Reactome and GO is shown in Table 1. Clearly, the schema of Reactome is more intricate and sophisticated than that of GO. This specialized structure of Reactome enables the capture of fine-grained details of cellular reactions. While GO and Reactome are equally able to capture atomic details, e.g., the location of a protein, its involvement in a complex, its role in mediating a molecular function, Reactome specifically supports joining and organizing these details to describe the molecular basis of an entire biological process.

**Table 1.** Comparison between Gene Ontology and Reactome

Gene Ontology (GO)		Reactome	
Category	Curated information	Category	Curated information
Gene product	Biochemical activity Subcellular location Biological objective	Gene product	Consumed in reaction Produced in reaction Catalyst activity Regulator of reaction Subcellular location Part of complex Part of polymer
		Reaction	Nature of reaction* Input of reaction Output of reaction Catalyst of reaction Part of pathway Preceding event Involved in disease Inferred from model organism Graphical representation
		Pathway	Reactions contained Associated GO term Involved in disease Graphical representation
Category	Annotations	Category	Annotations
Gene product	Associated with other gene products with suitable GO terms	Gene product	Associated with reactions (input/output/catalyst/regulator) Associated with other gene products (complex/polymer)
		Reaction	Associated with other reactions (preceding/following reactions) Associated with pathways (parent/other pathways)**
		Pathway	Associated with other pathways (parent/other pathways)**
Category	Annotation model	Category	Annotation model
Gene product	Manual annotation from scientific literature combined with electronic annotations	Gene product Reaction Pathway	Manual annotation from scientific literature (humans)***

*The three main reaction types in Reactome are binding, transformation (post-translational modifications, translocations, etc) and dissociation.

**Sub-pathways/reactions in Reactome can be shared by different pathways allowing the capture of crosstalk information.

***Pathways/reactions for non-human species are electronically predicted based on orthology analysis.

The systematic curation of autophagy mechanisms in GO [4] has resulted in the annotation of participating proteins, associations between these proteins and new terms that define various steps of the process. For instance, the serine/threonine-protein kinase, MTOR (mechanistic target of rapamycin kinase), is annotated with three autophagy-related GO terms, namely “negative regulation of autophagy”, “regulation of macroautophagy” and “negative regulation of macroautophagy”. A user may not be able to gain insight into the mechanistic interactions and crosstalk involving MTOR in the autophagy process. Reactome provides categories in addition to proteins, namely reactions and pathways, that can represent the complex biology of autophagy. Reactions and pathways show the interactions among proteins and are interconnected to represent crosstalk. Following the previous example, in Reactome the MTOR protein is shown to participate in five reactions in the macroautophagy pathway as a part of five distinct complexes in the lysosomal lumen. Reactions describe the role of MTOR in ULK1 complex phosphorylation and GTP hydrolysis. Reactome makes it possible to trace non-autophagic reactions and pathways that MTOR is involved in, making explicit molecular links between these pathways and autophagy.

### Hierarchically structured autophagy pathways

Pathways in Reactome are hierarchically structured in a biologically meaningful way and co-ordinated with the functional categorization of the biological process component of the Gene Ontology. The need for a well-defined pathway structure is critical to understand the autophagy process with all its molecular complexity spanning across multiple cellular compartments.

**Figure 2.**
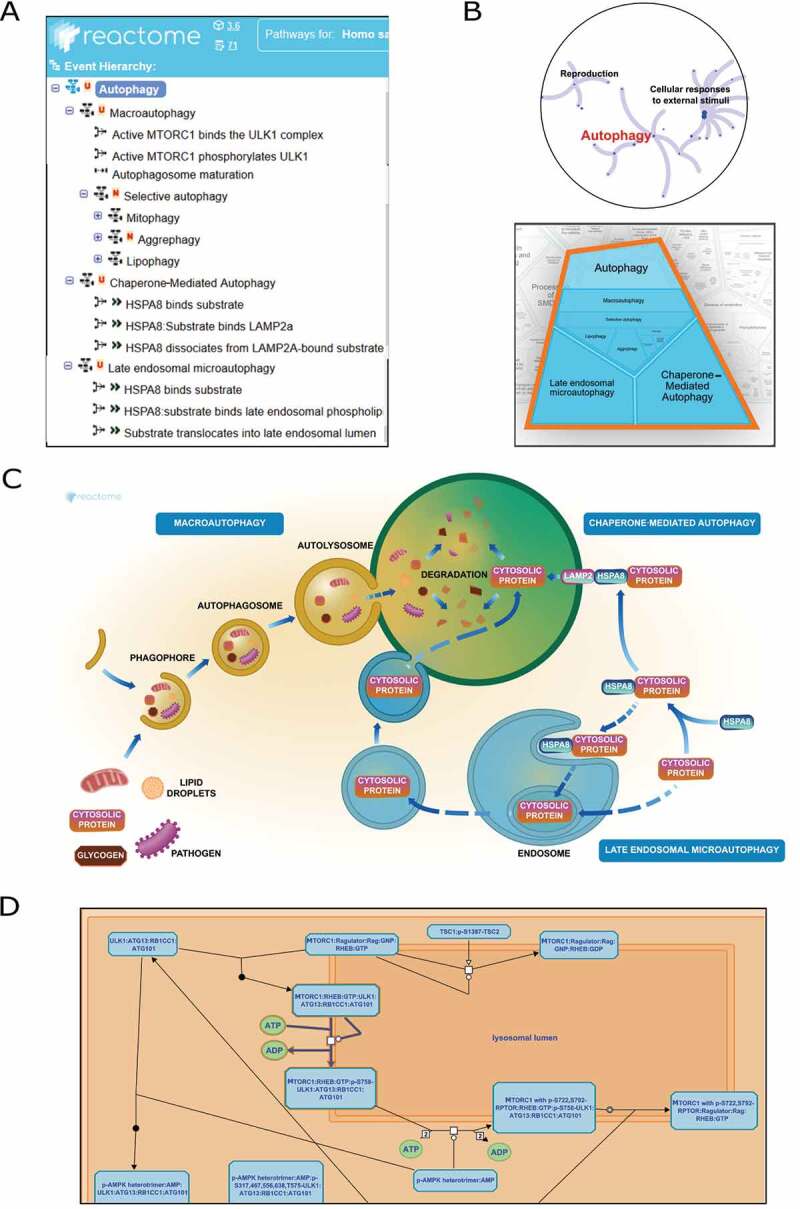
Representation of autophagy pathway in Reactome (A) Systematic organization of sub-pathways and reactions (B) Overview of the pathway designed as fireworks-styled and Voronoi diagrams (C) Textbook-styled diagram of the process (D) Detailed molecular reactions presented in SBGN format

Autophagy is a dynamic process with vital physiological consequences for the cell. It was important to create a separate top-level pathway for autophagy to account for the various interconnected events occurring over several cellular compartments. Three common forms of autophagy are widely reported, namely macroautophagy (MA), chaperone-mediated autophagy (CMA), and microautophagy (MI) [6,7]. In alignment with this classification, we created three corresponding sub-pathways under autophagy. MA primarily involves the engulfment of cytoplasmic cargo and the formation of the autophagosome, which eventually fuses with the lysosome. The CMA pathway involves the molecular chaperones that target and guide cytoplasmic material into the lysosome. The late endosomal MI pathway contains events that degrade cytoplasmic targets with the help of chaperones or membrane invaginations. Several forms of MA can be distinguished by the specific cargo (organelles/proteins); each form recruits cargo to the phagophore for eventual lysosomal degradation [8]. Each of these forms of selective autophagy also has cargo-specific features and is named according to its target: mitophagy for mitochondria degradation and lipophagy for lipid droplet degradation [9]. In Reactome, we placed different selective autophagy processes under a parent pathway: “Selective autophagy”, which in turn is a child of the MA pathway (Figure 2A). Users can efficiently navigate from a few top-level pathways to more intricate sub-pathways gaining knowledge about the process progressively.

The Reactome autophagy event hierarchy aligns well with the GO hierarchy of autophagy [4] (Table 2). The top-level pathway is autophagy with three children terms: MA, CMA, and late endosomal MI. MA has a child pathway termed “Selective autophagy” that includes mitophagy, lipophagy and aggrephagy.

**Table 2.** Classification tree similarity of autophagy process between Reactome and GO

Reactome hierarchy			
Autophagy (R-HSA-9612973)	MA (R-HSA-1632852)	Selective autophagy (R-HSA-9663891)	Mitophagy (R-HSA-5205647)
			Lipophagy (R-HSA-9613354)
			Aggrephagy (R-HSA-9646399)
	CMA (R-HSA-9613829)		
	Late endosomal MI (R-HSA-9615710)		
GO hierarchy [4]			
Autophagy (GO:0006914)	MA (GO:0016236)	Selective autophagy (GO:0061912)	Lipophagy (GO:0061724)
			Aggrephagy(GO:0035973)
	CMA (GO:0061684)		
	Late endosomal MI (GO:0061738)		
	Mitophagy (GO:0000422)		

### Curated information and external references

Well-established molecular mechanisms of cellular autophagy were manually extracted from the literature, curated, and annotated in Reactome (Table 3). Reaction details, such as input/catalyst/output molecules, reaction type, preceding reactions and experimental species, were captured during this process. Reactions were linked together based on preceding and following steps to generate pathways. Specific autophagy processes, such as mitophagy and lipophagy, were categorized under the “selective autophagy” pathway within MA. Sub-pathways in autophagy were interconnected via shared molecular entities and reactions. This curation strategy allows easy and quick inclusion of novel data as new molecular mechanisms are identified. To facilitate data access, Reactome reactions/pathways are linked to external data sources such as Ensembl [10], Gene Ontology [11,12], PubMed, ChEBI [13], UniProt [14], OMIM (https://omim.org/), IntAct [15] and Complex Portal [16]. This allows users to efficiently navigate between the different resources to mine information.

**Table 3.** Manually curated and annotated reactions in autophagy in Reactome version 71

			Gene Ontology annotations			
Pathways	Total entities*	Total reactions	Cellular components	Molecular functions	Biological processes	Total literature references
Macroauto-phagy	79	39	Cytosol/endosome/lysosome/endoplasmic reticulum/phagophore/autophagosome	kinase/GTPase/phosphatase/transferase/endopeptidase/ligase	Macro- autophagy	35
Chaperone-mediated autophagy	27	19	Cytosol/lysosome	-	Chaperone mediated autophagy	16
Late endosomal microauto- phagy	5	3	Cytosol/endosome	-	Late endosomal microauto- phagy	5
Mitophagy	45	15	Cytosol/mitochondria	Transporter/transferase/kinase/phosphatase	Mitophagy	17
Lipophagy	9	5	Cytosol/lipid droplet	-	Lipophagy	5
Aggrephagy	26	15	Cytosol/microtubule	-	Aggrephagy	12

*entities include proteins, chemicals, complexes and polymers.

### Enriched visualization of autophagy processes

Reactome graphical representations of reactions and pathways are designed to present information in a user-friendly style. There are three different levels of visualization of the autophagy pathway hierarchy in Reactome (Figure 2), each serving a unique purpose.

1. Pathways overview: An interactive panel provides information on the hierarchy of the autophagy process showing all the child pathways and interconnections among them. Two different display formats are available – the “fireworks” style, where the autophagy pathway hierarchy is represented as a concentric ring of sub-pathways and the “Voronoi” style where autophagy sub-pathways are displayed with areas proportional to the sizes of the sub-pathway contents in Reactome (Figure 2B).
2. Textbook-style figures: Biologists are used to seeing cellular pathways as graphical figures that explain the process temporally and spatially. To accommodate this customary pathway view, Reactome provides user-friendly textbook-style overviews of pathways (Figure 2C). The graphical illustration of the autophagy process showcases the mechanistic summary of MA, CMI and MI. This illustration is interactive and allows the user to navigate and select sections of the process.
3. Standard graphical representations: With some exceptions, Reactome follows the community standard Systems Biology Graphical Notation (SBGN) convention [17]. The SBGN representation of the autophagy process in Reactome provides elaborate interactive information on reactions, adjacent reactions and participating molecules, among other details (Figure 2D).

### Autophagy interaction with other pathways

Biological pathways, though functionally categorized, do not act independently and in an isolated fashion. Multiple input stimuli affect several interconnected pathways that generate an integrated response. An important aspect of pathway crosstalk mechanisms are shared components that couple different pathways. Reactome allows users to investigate components that are shared by various pathways in different compartments and forms.

**Figure 3.**
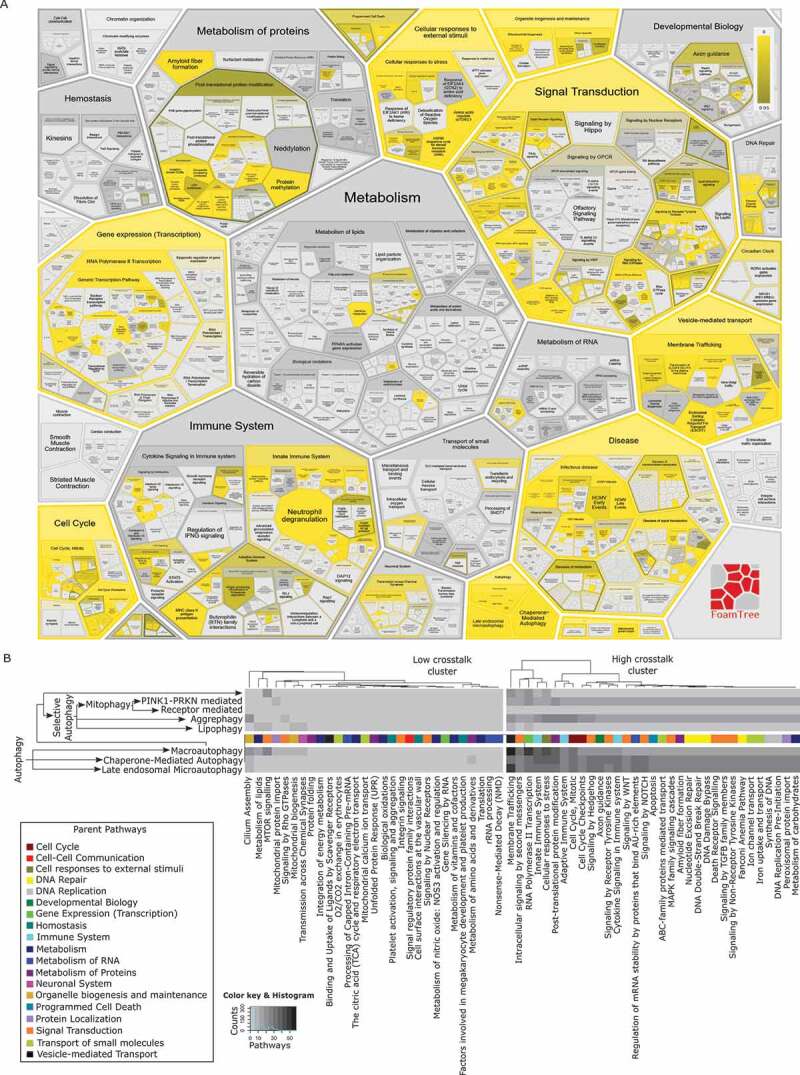
Autophagy interaction with other Reactome pathways based on shared proteins. This figure was generated by running a Reactome pathway analysis with the 127 proteins from the “Autophagy” top-level pathway and its child nodes. (A) Enrichment of autophagy proteins in Reactome pathways (B) Clustered heatmap of autophagy proteins present in other pathways (first level children)

Components of the autophagy pathway have a rich and diverse interaction profile with several vital cellular processes in Reactome. We performed a pathway enrichment analysis of autophagy proteins to identify autophagy-couples processes (Figure 3A). Significantly enriched pathways include innate and adaptive immune systems, cellular response to stress, membrane trafficking and several signal transduction networks. This finding supports our evolving understanding of the role that autophagy plays in processes, such as nutrient sensing [18], membrane trafficking [19] and immune system regulation [20]. Further, we analyzed individual autophagy mechanisms (MA, CMA and late endosomal MI) and observed distinct profiles of shared proteins for each autophagy subtype (Figure 3B).

### Data analysis tools

In addition to being a reliable database of cellular pathways, Reactome provides several tools to analyze user data against curated information. Pathway enrichment analysis is a common approach that provides mechanistic insight into gene lists from high-throughput experiments. Reactome provides a user-friendly interface to perform pathway enrichment analyses, allowing users to vary several parameters of the analysis. A second analysis tool in Reactome overlays expression data from omics studies across curated reactions/pathways. This feature lets a user visualize gene expression profiles as activated or repressed pathway profiles. Users can compare curated information in humans with a range of other species using a predictive orthology-based approach with the Species Comparison tool. Manually curated cellular reactions/pathways in humans are used to computationally infer reactions in fourteen evolutionarily divergent eukaryotic species with high quality, whole-genome sequence data. Furthermore, it is also possible to analyze pathways in a tissue-specific fashion based on expression data integrated within Reactome.

To illustrate the significance of Reactome in the field of autophagy research, we considered the study by DeJesus R et al. [21] and investigated what value Reactome adds to this work. SQSTM1 (sequestosome-1) is a receptor protein that is involved in recruiting ubiquitinated cargo into forming autophagosomes. DeJesus R et al. use a genome-wide CRISPR screen to identify novel regulators of SQSTM1, including several autophagy machinery components. Specifically, DeJesus et al. knockout genes using CRISPR-Cas9 techniques and measure SQSTM1-GFP signals in cells. GFP low vs. GFP high cells represent the degradation of SQSTM1-GFP, indicating the activation of autophagy, while GFP high vs. GFP low cells represent the accumulation of SQSTM1-GFP, indicating inhibition of autophagy. The top 100 SQSTM1 activating/repressing genes are subjected to an enrichment analysis using gene sets from various resources, including Reactome, NetPath, KEGG, Gene Ontology, Wikipathways and BioCarta, to identify modulated biological processes (Figure 2 in [21]). Subsequently, the authors empirically validate a novel SQSTM1 modulator UFM1 (ubiquitin-fold modifier 1) by uncovering the mechanism of regulation.

**Figure 4.**
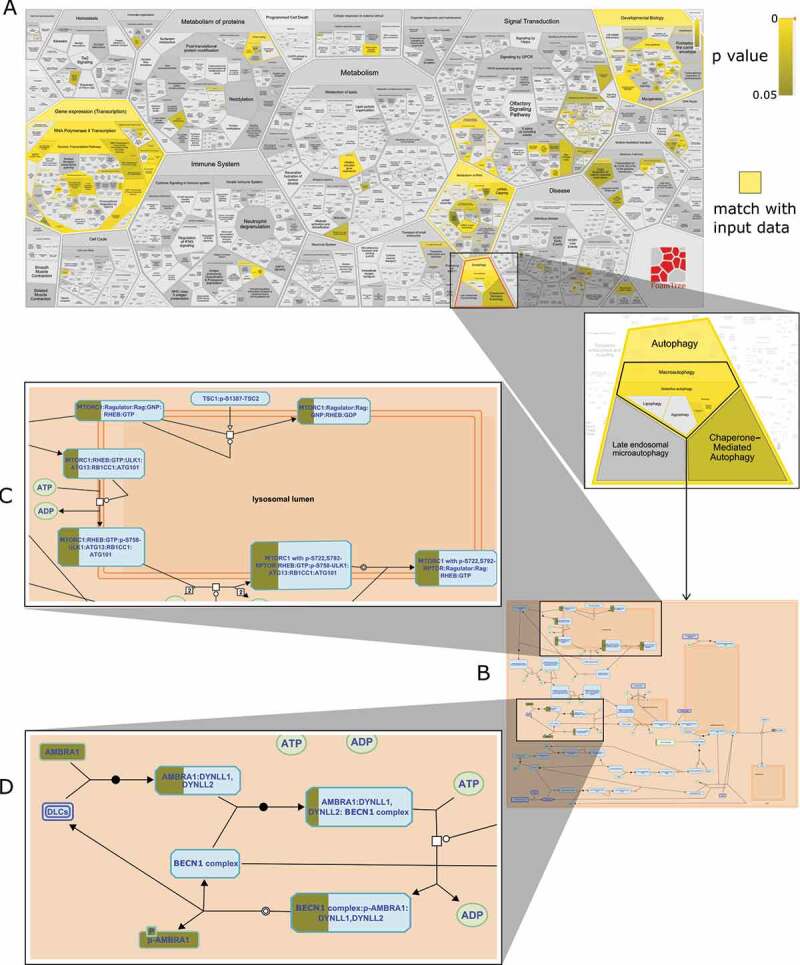
Reactome enrichment analysis of top 100 genes from GFP low vs. GFP high (autophagy activation) data from [21] (A) Distribution of 100 genes across Reactome pathway – yellow indicates matched entities and gradient represents significance (p-value) (B) Overlap of CRISPR-knocked out genes on the macroautophagy pathway (C) Macroautophagy inhibitory mechanisms – MTOR phosphorylation of ULK1 is significantly matched (D) Macroautophagy inhibitory mechanisms – AMBRA1 sequestering BECN1 complex is significantly matched

To reanalyze data from DeJesus R et al., we performed an enrichment analysis in Reactome using the top 100 SQSTM1 degrading/accumulating gene knockouts from CRISPR-Cas9 screening experiments (Figure 4). We observed that similar pathways were enriched in our analysis, as were identified in the work of DeJesus R et al. (Table 4). Autophagy and MA, in particular, emerged as the highly enriched pathways in the analysis (Figure 4A). We observed two distinct patterns of autophagy mechanisms highlighted in Reactome from the data [21].

**Table 4.** Enriched pathways for top 100 SQSTM1-modulating genes

Top 100 genes (Low vs High: SQSTM1 degradation)		Top 100 genes (High vs Low: SQSTM1 accumulation)	
DeJesus R et al. analysis [19] (GO process)	Reactome analysis (Curated pathway)	DeJesus R et al. analysis [19] (GO process)	Reactome analysis (Curated pathway)
transcription initiation from *POLR2*/RNA polymerase II promoter	autophagy	autophagosome assembly	autophagy
gene expression	macroautophagy	macroautophagy	macroautophagy
EPH/ephrin receptor signaling pathway	CD28 co-stimulation	mitophagy	nonsense-mediated decay
macroautophagy	PPARA activates gene expression	nucleophagy	nonsense-mediated decay enhanced by the exon junction complex
regulation of transcription from *POLR2*/RNA polymerase II promoter	Regulation of lipid metabolism by PPARA/PPARalpha	cellular response to nitrogen starvation	receptor mediated mitophagy

With the low vs. high (autophagy activation) data, Reactome correctly highlighted the inhibitory steps in autophagy. Experimentally knocked out genes were involved in autophagy inhibition reactions. These steps primarily overlapped with two suppressive mechanisms of the MA pathway – MTOR phosphorylation of serine/threonine-protein kinase ULK1 and the binding of AMBRA1 (autophagy and beclin 1 regulator 1) with the BECN1 (beclin 1) complex (Figure 4B). MTOR complex 1 (MTORC1) phosphorylates and deactivates ULK1 under normal conditions. Upon nutrient starvation, the AMP-activated protein kinase (AMPK) complex is activated and triggers the dissociation of ULK1 from MTORC1 via phosphorylation. Subsequently, ULK1 initiates the autophagy process to respond to the starvation stimuli. The second mechanism involved the inhibition of BECN1 complex activity by sequestering it to the cytoskeleton with the help of AMBRA1 and dynein complexes. Upon stimuli, AMBRA1 is phosphorylated and dissociates from the BECN1 complex, which then triggers the autophagy process. Another highlighted mechanism is mitophagy where the data suggests a role for proteins in the translocase of outer mitochondrial membrane (TOMM)-translocase of inner mitochondrial membrane (TIMM) and casein kinase 2 (CSNK2) complexes. Elimination of TIMM-TOMM and CSNK2 complexes can trigger autophagy by preventing the import of the serine/threonine-protein kinase PINK1 into the inner mitochondrial membrane and blocking FUNDC1 phosphorylation, respectively.

Reactome correctly interpreted the high vs. low (autophagy inhibition) data by highlighting the activation steps in autophagy. Experimentally knocked out genes were involved in autophagy activation reactions. Activation mechanisms included ULK1 signaling, BECN1 complex reactions, and ATG proteins system within the MA and mitophagy pathways.

Genes that were not found in the enrichment analysis in Reactome are listed in Table 5. Proteins may be unavailable because they have not yet been curated in Reactome or their functions are still unknown. It may be worthwhile for researchers to validate the significance of these genes in modulating SQSTM1.

**Table 5.** Genes not found in Reactome enrichment analysis using top 100 SQSTM1-modulating genes

Top 100 genes (Low vs High: SQSTM1 degradation)	Top 100 genes (High vs Low: SQSTM1 accumulation)
*TOX4* *PPP1R2* *TFAP4* *SLTM* *UBR5* *EMC3* *SERGEF* *HYPK* *EIF1*	*SPEN* *BCAS3* *TOR2A* *ATMIN* *TLCD3B/FAM57B* *VPS33A* *NOLC1* *UFSP2* *SOX11* *KLHDC7A* *EFL1/EFTUD1* *PRRC2A* *YAE1/YAE1D1* *CXorf56* *IL17REL* *CCDC9* *SRRD* *CINP* *BBX* *HNRNPH3* *ATXN10* *CCDC130* *LTO1/ORAOV1* *BRD4*

Mechanistic knowledge of pathways provides useful insights into identifying critical molecular events and predicting the consequences of perturbations. DeJesus R et al. investigate enriched Gene Ontology (GO) biological processes that modulate SQSTM1 and autophagy emerged as an important regulating module. This approach is useful to get an overview of the SQSTM1-regulating pathways. However, there is little information on the molecular interactions in the autophagy pathway and how identified genes fit within the reactions of the pathway. Reactome analysis, on the other hand, sheds light on reactions of autophagy pathways by graphically laying down molecular mechanisms and projecting the user gene list over reactions (Figure 4C). Users can visually infer the overlap of empirically identified genes with components of the autophagy machinery in Reactome. Such graphical layouts of pathways can be very useful to decode the processes triggered by input gene lists.

Molecular events in Reactome are extensively cross-referenced to other biological resources to facilitate effective analysis. In addition to providing insight into enriched reactions within autophagy, enrichment analysis of DeJesus R et al. data in Reactome also links users to different aspects of the pathway components. Examples include studying protein sequences and structures, investigating other interactors of autophagy proteins or retrieving reference articles for more information. This exercise may allow researchers to better understand their empirical data and assist in planning future experiments.

### Programmatic access to data

It is essential to access and analyze data in Reactome computationally. To this end, an application program interface (API) allows users to programmatically work with Reactome. The API is based on the Representational State Transfer (REST) protocol for efficient service and facilitates data retrieval via HTTP requests.

**Figure 5.**
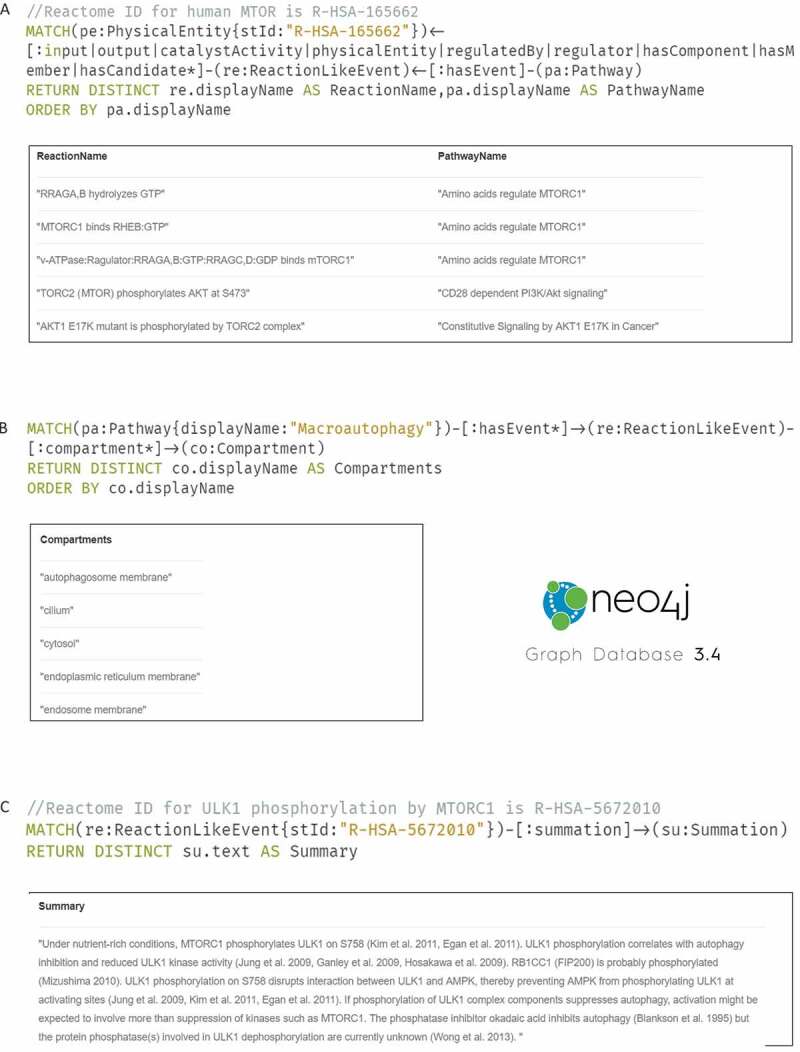
Reactome querying using Cypher and Neo4j (**A**) Extracting the reactions/pathways involving MTOR protein (**B**) Retrieving cellular compartments involved in macroautophagy (**C**) Obtaining a human-readable summary on ULK1 phosphorylation by MTORC1

As an alternative representation, Reactome is available as a Neo4 j graph database. Users can intuitively interact with Reactome graph database in Neo4 j with the help of Cypher query language. Users can ask questions such as “What are the reactions and pathways that involve MTOR in Reactome?” (Figure 5A) or “What cellular compartments are involved in MA?” (Figure 5B) or “How does MTORC1 phosphorylate ULK1 what literature evidence supports this?” (Figure 5C).

### Training materials in Reactome

Hosting a broad spectrum of biological information with several data analysis tools, thorough documentation and a detailed tutorial are essential. The Reactome website provides an extensive user guide with step-by-step instructions on how to use the service. Short videos providing a virtual tour of Reactome are also available. Elaborate training materials on the service are also available on the European Bioinformatics Institute website [22–26]. For more specific queries, we support users via e-mail at help@reactome.org. Reactome aims to engage the community in curation and reviewing activities.

## Discussion

Autophagy has been an elusive and surprising research area in molecular biology with its history going back as far as 1955 when the discovery of the lysosome [27] was applauded with a Nobel Prize in Physiology or Medicine. After this, a few decades would pass, during which the field got little attention from the scientific community. The discovery of 15 autophagy-related proteins (ATGs) [28] in 1993 revived community interest and paved the way for autophagy into mainstream research. This effort was once again acknowledged with a Nobel Prize in Physiology or Medicine. Today, we know a lot about the molecular mechanisms and physiological significance of autophagy and this knowledge is increasing still. Despite playing a central role in cellular homeostasis, autophagy is also linked to various pathophysiologies, including cancer development and progression, neurodegenerative diseases, infection and inflammation [29].

Autophagy is an intricate mechanism, involving a multitude of cellular components and compartments with several physiological implications. Decades of research in the field has revealed vital information about the molecular interactions and their consequences. As this cumulative knowledge has grown, it has become challenging to compile and comprehend.

Reactome empowers researchers to search and analyze information by serving as a knowledgebase for the autophagy process. Molecular events in autophagy are manually curated from literature by qualified professionals and peer-reviewed by experts in the field. Consequently, the data in Reactome is highly reliable and accessible. Manual curation and review of cellular events is labor-intensive, time-consuming and may be prone to data selection bias. We are making efforts to address these concerns. Currently, we are working on an online reviewer system that will allow experts in the field to visit the website and review relevant curated information with minimal human interaction required. With our ongoing collaboration efforts with other bioinformatics resources, we aim to transfer curated knowledge into Reactome. As a future possibility, we are exploring text-mining and machine learning as potential tools to assist the curation process.

The infrastructure facilitates the addition of new information about pathway mechanisms as it becomes available, allowing for the revision and updating of pathways when necessary. Autophagy curation is an ongoing effort with regular addition and modification of reactions in line with the evolving science. Reactome allows new information to be naturally added to the existing structure of the autophagy pathway, thereby maintaining the current knowledge, as well as allowing for extension. As the autophagy pathway in Reactome expands with time, users can benefit from this comprehensive knowledge with enhanced crosstalk information and better analysis (inferred from Figure 1).

Autophagy reactions are richly annotated and cross-referenced to external resources providing a comprehensive picture of the field. Reactome data is extensively mapped to several databases and *vice versa*, resulting in a highly inter-operable interface for users. We collaborate with external resources to produce synergistic and emergent value. For example, standard Gene Ontology curation captures features of individual gene products, while Reactome focuses on the relationships among these gene products in the context of whole reactions and the causal connections among these reactions. The recent development of GO-CAM models [30] and our collaboration with them to export Reactome reaction annotations into GO-CAM format helps to bridge this knowledge gap.

Reactome allows users to perform various common pathway analysis operations via a user-friendly interface in a few steps. Autophagy mechanisms in non-human model organisms can also be studied via an ortholog-based prediction tool. Such analyses of autophagy pathways at the molecular level facilitate a user’s novel perspective for the empirical data. Reactome is intended to be a one-stop-shop to search and analyze information about molecular autophagic events and add value to research in the field.

Reactome aims to facilitate streamlined research by providing enhanced features to process biological knowledge. Users can exploit the RESTful API or Neo4 j service to retrieve and analyze complex information on autophagic reactions. Reactome provides a tool to predict the distribution of autophagy processes in different tissues based on RNA/protein expression profiles. The ReactomeFIViz (Reactome Functional Interaction Visualization) app allows researchers to perform various pathway and network-based data analysis, as described previously in [31]. User guides and tutorials provide comprehensive information on all of these features in Reactome.

Reactome is an evolving knowledge base that accommodates growing and changing information. With a quarterly update cycle, researchers can expect to find new information every three months. Novel discoveries in autophagy mechanisms can be incorporated in Reactome as they emerge. Users are free to use Reactome data (with citation) for research and publications as we have an open usage policy.

## Materials and methods

### Reactome interface

1. Interactive browsing: The complex nature of the data in Reactome demands an interactive interface allowing the user to filter relevant information or choose the desired level of detail. To this end, Reactome provides a highly interactive website for users to effectively access information. Pathways are structured hierarchically in Reactome following consensus ontology trees allowing intuitive navigation in a graphical interface that supports zooming and panning.
2. Data analysis tools: The website has a dedicated section for data analysis that allows users to perform pathway enrichment analysis, topological analysis, expression overlay analysis, species comparison, and tissue-specific analysis.
3. Programmatic access: Users can computationally extract/compare information in Reactome in bulk or use it in a bioinformatics pipeline. Reactome provides a REST-based content service and a Neo4 J graph database.
4. Documentation: Detailed documentation of the features and short tutorials are available on the website (https://reactome.org/userguide). This includes instruction on how to search, interpret screen layouts, and use the different tools in the interface. Users are encouraged to contact the team with any questions.

### Curation process

1. Manual curation from literature: Cellular reactions in Reactome are manually extracted from published scientific literature by curators. Curators scan literature for information on biological events, including but not limited to biochemical reactions, binding and dissociation, and post-translational modifications. Events are consistently named according to internal conventions, identifying key reaction participants and their fates [32]. Individual reactions are linked to preceding reactions to generate chains of events that result in pathways. Every reaction in Reactome is validated with literature references enabling users to access the paper for further details. Curators add a free text summation of each reaction with citations providing an easily comprehensible overview of the event in context of the pathway.
2. Internal and external expert review protocol: Several measures are taken to ensure a high standard in data accuracy and consistency in Reactome. Information from scientific papers is processed by well-qualified curators with post-graduate degrees. This work then passes through a first round of internal review by the Reactome curation team checking for consistency and compatibility with Reactome standards. This version is then submitted to an external domain expert to validate the scientific accuracy and consensus in the field. In most cases, external domain experts are involved early on in the process. When the curated information successfully completes the review process, it goes through computational quality assurance checks before it is prepared to be released on the website. To encourage community engagement in Reactome, we provide digital object identifiers (DOIs) for Reactome pathways and credit external authors and reviewers using an ORCID-based system to document their contributors [33].
